# Acute cough: a diagnostic and therapeutic challenge

**DOI:** 10.1186/1745-9974-5-11

**Published:** 2009-12-16

**Authors:** Peter V Dicpinigaitis, Gene L Colice, Mary Jo Goolsby, Gary I Rogg, Sheldon L Spector, Birgit Winther

**Affiliations:** 1 Albert Einstein College of Medicine and Montefiore Medical Center, Bronx, NY, USA; 2 George Washington University School of Medicine, Washington, DC, USA; 3 American Academy of Nurse Practitioners, Augusta, GA, USA; 4 UCLA School of Medicine, Los Angeles, CA, USA; 5 University of Virginia Health System, Charlottesville, VA, USA

## Abstract

**Background:**

Acute cough is one of the most common complaints prompting patient visits to healthcare professionals. Despite the broad repercussions of acute cough on patient quality of life, school and work productivity, and public health resources, research on this condition is minimal, as are the available treatment options. Many patients use over-the-counter medicines, which are often ineffective for symptom relief. Some therapies may achieve antitussive activity, but at the expense of unpleasant or intolerable side effects.

**Unmet needs:**

When considering the treatments currently available for the management of acute cough, the multiple limitations of such treatments are quite apparent. Most of these treatments lack clinically proven efficacy and reliability to support their use. This reinforces the need for the generation of quality scientific data from well-performed clinical trials. Hopefully, the result will be the development of safer, more effective and more reliable therapeutic options in the management of acute cough.

**Cough assessment and management:**

Acute cough can be due to a variety of causes, and it is worthwhile to consider these pathogenic factors in some detail. It is also important to be familiar with the effects that acute cough has on patients' quality of life, work productivity, and the healthcare system; proper awareness of these effects may contribute to better understanding of the social impact of cough. In reference to the available treatments for the management of acute cough, adequate knowledge of the type of over-the-counter and prescription products in the market, as well as their mode of action and advantages/disadvantages, may provide expanded pharmacotherapeutic opportunities and facilitate better clinical decisions. However, due to the drawbacks of current treatment options, ideas for future cough management and newer products need to be considered and tested.

**Conclusion:**

In view of the socio-economic impact of acute cough and the limitations of available treatments, a renewed interest in the management of acute cough needs to be encouraged. The current strategies for acute cough management need to be reassessed, with a focus on developing new, reliable products and formulations with proven efficacy and safety.

## Review

### Introduction to acute cough

Acute cough is one of the most common symptoms for which patients seek medical attention and spend healthcare dollars [1], the most common new presentation in primary care [2], and the most frequent reason for visits to hospital-based outpatient clinics [3]. In the USA, acute cough accounted for 26 million office visits in 2004 [4]. In the vast majority of cases, acute cough is due to acute viral upper respiratory tract infection (URTI), i.e., the common cold.

Notably, over the past 50 years, pediatric immunization has dramatically decreased pediatric pertussis cases, from 157 to less than 1 per 100,000 persons [5], but has not decreased the incidence in adults. In fact, during the 1990s, the number of pertussis cases in adolescents and adults more than doubled in the USA and Canada [6]. In a 2.5-year study, the incidence of pertussis in 2,444 healthy people, aged 5-65 years, ranged from 370-450 cases per 100,000 persons per year. Extrapolated to the USA population, nearly a million pertussis cases occur per year [7].

By definition, acute cough is one lasting <3 weeks, sub-acute cough lasts 3-8 weeks, and chronic cough lasts >8 weeks [8]. Most acute coughs raise minimal concerns among health practitioners as they are generally caused by URTIs, usually have a short duration, and are self-limited. However, acute cough may be a symptom of a serious underlying condition, such as pneumonia, acute pulmonary embolism, pulmonary edema, or lung cancer. It is the most common symptom associated with acute exacerbations and hospitalizations with asthma and COPD (Table 1) [2].

**Table 1:** Causes and estimated frequencies of acute cough in the adult [1]

**Common**	**Less common**
Common cold	Asthma
Acute bacterial sinusitis	Congestive heart failure
Pertussis	Pneumonia
Exacerbations of COPD	Aspiration syndromes
Allergic rhinitis	Pulmonary embolism
Environmental irritant rhinitis	

Reproduced with permission from: Irwin RS et al. *Chest *1998, 114:133S-181S.

Despite the significance of cough in clinical practice, the clinical interest and research efforts in the study of cough have been historically sparse [9], and there have been no new antitussive treatments in the past 50 years [10]. However, recent years have seen a heightened scientific, clinical, and pharmaceutical interest in cough, along with a steady increase of publications on this subject [9,11]. Recent years have also witnessed the release of evidence-based guidelines for the diagnosis and management of cough from the American College of Chest Physicians (ACCP) [12], the European Respiratory Society [13], the British Thoracic Society [2], and the Japanese Respiratory Society [14].

This article reviews the limited research in acute cough, the impact of acute cough on quality of life and health economics, current treatment options, and potential treatments to satisfy unmet needs in the management of this common ailment.

### Pathology

Cough is a forced expulsive maneuver, usually against a closed glottis, and is associated with a characteristic sound [2]. In most healthy individuals, cough is an important natural reflex and defense mechanism that helps to clear excessive secretions and prevent foreign matter from entering the airways. However, when the respiratory system becomes compromised, cough can become excessive, nonproductive, disturbing to the patient, and potentially harmful [15].

Although many factors can induce acute cough, URTIs, usually of viral origin, are the most common [16]. While the common cold is the most frequent cause, other factors causing acute cough include viral rhinosinusitis, acute bronchitis/sinusitis, and acute exacerbation of COPD [1]. Acute cough can be induced from the upper airways by rhinovirus, which primarily infects the nasopharynx, and can be cultured from the nasopharynx for up to 3 weeks [17]. Patients on anti-inflammatory treatment may shed the virus for a longer time [18]. Patients with asthma may also shed rhinovirus from the lower airways. Of note, cough reflex sensitivity, as measured by capsaicin inhalation cough challenge, is transiently enhanced during an URTI [19,20].

A study of children aged 5-12 years found that symptoms of rhinovirus colds differ in children and adults. In symptom diaries completed with the assistance of parents, children more frequently report cough during the first 5 days of illness, whereas adults primarily report nasal discharge, persisting only through day 4. Rhinovirus-induced acute cough peaks at about 40% in adults on days 3-5 and drops to about 20% by day 10. In children, cough peaks on day 2 at over 70% and is still reported in more than 40% of children through day 9, when it finally falls below 40% [21].

Cough is commonly triggered when sensory cough-inducing receptors in the respiratory tract are stimulated by mechanical or chemical irritation [1]. Mechanical irritation can be due to factors ranging from inhaled irritants (e.g., dust, dandruff [22], smoke), to excess and tenacious mucus, and somatic conditions such as infections. Particle exposure to common allergens (e.g., dust mites, animal allergens, and pollen) can induce cough after eliciting upper or lower respiratory tract reactions, such as allergic rhinitis and asthma [23]. Exposure to chemicals, such as chloramines in swimming pools, may also affect a large number of individuals [3,24]. Cough can also be chemically induced by angiotensin-converting enzyme inhibitors. This typically nonproductive cough is associated with an irritating, tickling or scratching sensation in the throat, and will disappear or substantially improve within 4 weeks of discontinuing the drug [1].

### Quality of life and economic impact

Acute cough can be very disruptive to patients' well-being and adversely affect family members and co-workers as well. Most patients seek medical attention because of concerns or complications related to the cough syndrome, such as worries about the intensity of cough symptoms, perceptions of fatigue associated with cough, feelings of self-consciousness, and symptoms of sleep deprivation, hoarseness, musculoskeletal pain, sweating, and urinary incontinence in women. Some serious complications associated with vigorous coughing may require prompt assessment and treatment, e.g., cough-related syncope, cardiac arrhythmias, pneumothorax, splenic and venous ruptures, seizures, loss of consciousness, and disruption of surgical wounds and intravascular catheters [1].

Other factors prompting patients to seek professional healthcare are socially avoidant behaviors, vomiting, depression, and excessive perspiration [1]. Some patients experience symptoms for many weeks, even years, before they seek medical help and, in some cases, the patient's relatives or partner initiates the medical referral [25]. The potential benefits of treating cough early could be preventing the vicious cycle of cough perpetuating cough [26] and decreasing the infectious spread of viruses by decreasing cough.

A study investigating the impact of acute cough on health-related quality of life revealed that cough had an adverse effect on well-being in both men and women. However, significantly more women complained of urinary incontinence and exhaustion, whereas significantly more men noted a concern of cancer and having to make lifestyle changes as a result of their cough [27]. Table 2 summarizes the most common adverse symptoms associated with cough [25].

**Table 2:** Adverse symptoms associated with cough [25]

**Physical**	**Psychological**	**Social**
Syncope	Depression	Relationship tensions
Vomiting	Anxiety	Fear of public places
Chest pain	Embarrassment	Avoidance of social events
Hoarse voice	Fear of serious illness	Interference with work
Headache	Frustration	Interrupt telephone calls
Incontinence		Interrupt meals
Hernia		
Sleep deprivation		
Lethargy		

Reproduced with permission from: Brignall K et al. *Lung *2008, 186 Suppl 1:S55-S58.

URTIs also impose a significant economic burden. Studies done in Europe and Australia have shown that the healthcare costs of acute cough and URTIs in children are substantial [28,29]. Similarly, an analysis of hospital admissions in the UK from 1990 to 2005 documented a considerable increase in hospitalizations in the elderly from respiratory episodes in winter, with acute bronchitis (for which the main symptom is acute cough) being the primary and most consistent reason for the hospitalizations [30].

The annual cost of OTC cough medicines in the USA is estimated to be in the several-billion-dollar range, despite a lack of efficacy for many of these medicines [31]. In addition to the direct and indirect healthcare costs of acute cough, there is a significant morbidity with cough syndromes that imposes additional burdens and healthcare expenditures [31,32]. Considering the high socioeconomic impact of reduced productivity associated with acute-cough syndromes, URTIs are one of the most common reasons for work and school absenteeism [32], and there is a cascade of productivity losses by caregivers when a child is sick [28].

A study to quantify the cost of viral respiratory tract infections in the USA found that when survey results of 4,051 respondents who experienced cough in the past year were extrapolated to the population, the total economic burden approaches $40 billion annually. This includes $17 billion in direct healthcare resource (medications, medical services) costs and $22.5 billion in indirect costs (productivity losses), per year [33].

### Acute cough management

Appropriate management of acute cough includes sequential evaluation and treatment of the likely causes of cough, using both diagnostic tests and appropriate empiric therapy. The most important initial decision is to determine whether the cough is a sign of a serious, potentially life-threatening condition--such as pneumonia, pulmonary embolism, congestive heart failure, asthma, COPD, bronchiectasis, or lung cancer--or, as is commonly the case, a result of the common cold or exposure to an environmental allergen or irritant [34].

Treatment of acute cough caused by viral URTIs tends to be symptomatic, with the aim of suppressing the hypersensitized cough reflex while the underlying cause is cleared naturally. A medical history and physical examination are usually sufficient to determine whether the acute cough is due to a non-life-threatening URTI, a lower respiratory tract infection, exacerbation of an existing condition, or an upper airway cough syndrome [34].

**Figure 1:**
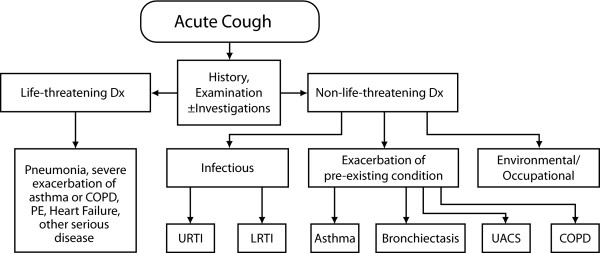
**Algorithm for assessment of acute cough in patients ≥ 15 years of age (adapted with permission from Irwin et al., 2006) **[8]. (URTI = upper respiratory tract infection; LRTI = lower respiratory tract infection; UACS = upper airway cough syndrome; COPD = chronic obstructive pulmonary disease).

In some cases, acute cough may be indicative of a serious illness, requiring further investigation. An acute cough that is productive may be a sign of acute bronchitis due to a lower respiratory tract viral infection such as influenza A, bacterial infection, or another condition that mimics acute bronchitis [34]. If a patient remains symptomatic despite evaluation and treatment for 8 weeks, the cough is considered chronic and the primary care clinician should consider referral to a specialist. An algorithm showing differential considerations during the assessment of acute cough is shown in Figure 1[8].

### Current OTC treatments

Most patients initially use OTC cough and cold medicines to relieve acute cough and other symptoms associated with URTIs. However, a Cochrane review of OTC cough medicines, based on randomized controlled trials in children and adults, failed to clearly demonstrate the effectiveness of these medicines. For example, two Cochrane-reported trials on adults with 356 participants compare antihistamine-decongestant combinations with placebo. One trial comparing loratadine/pseudoephedrine (5 mg/120 mg twice daily for 4 days) with placebo (n = 283) did not show statistically significant differences in cough scores reported in patient diaries between both groups [35].

The second trial (n = 73) compared dexbrompheniramine/pseudoephedrine (6 mg/120 mg) twice daily for 1 week) with placebo. The mean severity rank of cough on a scale of 0-4, obtained through a patient diary, was less in the active group (1.4) than in the placebo group (2.0) on days 3-5 (p ≥ 0.05) [36]. There was an increased severity of dizziness and dry mouth in the active drug group on days 5-7 and 2-10, respectively. The Cochrane review was inconclusive because the number of trials was small and often with few patients [37,38]. An overview of OTC and prescription cough medicines is given in Table 3.

**Table 3:** Over-the-counter and prescription medicines available for the therapy of acute cough

Type of product	Component	Available in combinations with	OTC or prescription; formulation
**Cough suppressant**	Benzonatate	-	Prescription; perles and capsules
	DXM polistirex	-	OTC; extended-release suspension
	DXM	Antihistamine: *promethazine*	Prescription; syrup
	Hydrocodone	Agent to discourage overdose: *homatropine*	Prescription; tablet and syrup
	Hydrocodone	Antihistamine: *chlorpheniramine*	Prescription; extended-release suspension and extended-release capsule
**Antihistamine**	Brompheniramine	-	Prescription; elixir and injection
	Brompheniramine	Cough suppressant + decongestant: *DMX + pseudoephedrine*	Prescription; syrup
	Chlorpheniramine	Cough suppressant: *codeine*	Prescription; extended-release suspension
	Clemastine	-	OTC or prescription; tablet and syrup
	Desloratadine	-	Prescription; tablet and syrup
	Desloratadine	Decongestant: *pseudoephedrine*	Prescription; extended-release tablet
	Dexbrompheniramine	Decongestant: *pseudoephedrine*	OTC; extended-release tablet
	Dexbrompheniramine	Decongestant + antipyretic: *pseudoephedrine + acetaminophen*	OTC; extended-release tablet
	Diphenhydramine	-	Prescription; injection, elixir and capsule
	Loratadine	-	OTC; tablet and syrup
	Loratadine	Decongestant: *pseudoephedrine*	OTC; extended-release tablet
	Promethazine	Cough suppressant: *codeine*	Prescription; syrup
	Promethazine	Cough suppressant + decongestant: *codeine + phenylephrine*	Prescription; syrup
	Promethazine	Cough suppressant + decongestant + antihistamine: *codeine + phenylephrine + triprolidine*	Prescription; syrup
**Expectorant**	Guaifenesin	-	OTC; tablet
	Guaifenesin	Cough suppressant: *DXM*	OTC; tablet
	Guaifenesin	Decongestant: *pseudoephedrine*	OTC; tablet

OTC = over-the-counter; DXM = dextromethorphan

Most OTC cough medicines are short-acting syrups in two basic categories: cough suppressants (antitussives) and expectorants. Suppressants attempt to dampen the cough reflex to normal levels when its intensity is in excess of what is required to defend the airways [39]. The most commonly used OTC suppressant is dextromethorphan (DXM), which is considered generally safe at recommended doses. However, it can cause hallucinations when taken in large doses. Products containing DXM are rapidly becoming substances of abuse in the USA [40].

Expectorants may be useful in cases of excessive mucus production, by increasing the volume of mucus and facilitating the removal of secretions by ciliary transport and/or cough [38,41]. The only FDA-approved expectorant in the USA is guaifenesin, which has an established and benign safety profile when used as directed. Although guaifenesin is not typically known as a cough suppressant, it has been shown to inhibit cough-reflex sensitivity in patients with URTI in whom cough receptors are transiently hypersensitive [42,43], and to reduce subjective measures of acute cough due to upper respiratory infections (URIs) [39,44].

Many OTC products offer combinations of centrally acting cough suppressants (e.g., DXM) and expectorants (e.g., guaifenesin), as well as combinations of either drug with analgesics, decongestants, and/or antihistamines. Effective antihistamines in combinations are first-generation agents, such as dexbrompheniramine and chlorpheniramine. Non-sedating newer-generation antihistamines are considered ineffective for reducing cough in patients with the common cold [16].

### Prescription treatment options

Prescription cough remedies usually contain higher doses of cough suppressant than expectorant agents, and are typically prescribed when OTC remedies have failed to relieve disruptive cough symptoms. Relatively few drugs have been shown to suppress acute cough by an action on mucociliary factors, and none has been shown to do so consistently [39].

Inhaled anticholinergic agents have had inconsistent effects on acute cough, and some of their adverse effects can present challenges in clinical management and adherence [39]. However, a recent study has demonstrated the ability of inhaled tiotropium to suppress cough reflex sensitivity in subjects with acute viral URI [45]. The clinical significance of this finding remains to be elucidated. Similarly, first-generation antihistamines (brompheniramine, chlorpheniramine, clemastine, etc.) share a number of adverse effects with anticholinergic agents and may induce drowsiness and gastrointestinal distress.

Although benzonatate, which is believed to work by decreasing the sensitivity of stretch receptors in the lung, is effective for temporary relief of cough, there have been reports of severe adverse reactions to this product, including bronchospasm, laryngospasm, and cardiovascular collapse. Seizures and cardiac arrest are possible following an acute ingestion [46].

Studies of opiates in acute cough due to URTIs have shown mixed results. Although the antitussive effects of codeine in patients with chronic bronchitis were established in small patient populations, and there have been no double-blind, placebo-controlled studies of codeine in cough due to acute bronchitis, it is reasonable to presume that codeine is effective under these circumstances [39]. When administered orally, codeine has a short duration of action, and up to 95% of a single dose is excreted within 48 hours.

Current ACCP guidelines do not recommend the use of peripherally or centrally acting cough suppressants for the treatment of cough due to URTIs, and discourage the use of OTC combinations for the treatment of acute cough due to the common cold, except for an older combination of a first-generation antihistamine plus a decongestant [39]. Patients with acute cough or upper airway cough syndrome can also be administered naproxen to help reduce cough symptoms [16].

### Discussion and future direction of cough management

While there are no established guidelines for when patients should seek medical attention for cough, the authors believe that adults should see a healthcare professional after 8 weeks, at which time the cough is considered chronic. Prior to that, a visit to a professional will depend on cough severity, patient discomfort, and impact on quality of life. However, medical attention should be sought immediately if acute cough is accompanied by certain symptoms that may indicate serious underlying problems: cough with fever and purulent sputum (pneumonia), cough with significant dyspnea (pneumonia, pulmonary embolism, congestive heart failure), and cough with hemoptysis (pneumonia, active TB, endobronchial lesion). Clear guidelines and public education on when patients should seek medical attention for cough, as well as an improved patient-reported cough severity measure, and consideration of the quality-of-life impact in cough management are warranted.

Historically, there has been a dearth of scientific evidence and research in acute cough treatment. When it may be preferable to suppress viral-induced acute cough and when it may be preferable to enhance it utilizing expectorants has not been adequately investigated. Currently available cough-suppressant therapy is limited by a paucity of effective agents and/or their unacceptable side effects. There is also a lack of clinically useful tools to measure the effect of cough suppressants and drugs that address symptomatology.

Most current treatments are liquid formulations, which share common problems with all medicines not dispensed in tablet form, including difficulties with precise measuring of doses and the common practice of exceeding recommended doses, which can lead to significant unintended complications. Storage and transportation are other relevant disadvantages of liquid formulations, especially when traveling.

The ideal treatment for acute cough would not only have a well-established safety profile, but also provide rapid and long-acting relief, with sufficient effectiveness to allow patients to sleep throughout the night. In the future, longer-acting formulations of cough-suppressing agents using extended-release technology to deliver sustained relief, or existing agents used in novel combinations may play an important role in developing more optimal treatments for acute cough. Current research is also investigating alternative cough suppressants that may have improved side-effect profiles. These include large-conductance calcium-activated potassium channel openers and agents selectively targeting various receptors (e.g., vanilloid receptor antagonists, selective opioid or opioid-like receptor agonists, tachykinin receptor antagonists, endogenous cannabinoid type-1 receptor agonists and antagonists, 5-hydroxytryptamine receptor agonists) [47].

## Conclusions

Acute cough is a serious problem that has an adverse impact on the well-being of patients, families, and caregivers, and on health economics. The clinical morbidity and quality-of-life and economic issues associated with acute cough warrant increased attention to this common syndrome.

Most current treatments for acute cough lack evidence-based proof of efficacy to support their use. Many are short-acting liquid formulations, and many contain anticholinergics, first-generation antihistamines, or DXM. Guaifenesin, the only available OTC expectorant with antitussive effects is short acting, but more recently long-acting guaifenesin formulations and combination products have been launched. Codeine, the most common prescription opiate antitussive, is only available in a short-acting form and requires frequent daily dosing. Treatment with a prescription medication is frequently necessary to control disruptive cough symptoms, even if the underlying cause of symptoms--the acute viral infection--is self-limiting.

There is a need for a reliable, longer-acting formulation in solid form that can safely and consistently deliver relief of cough for extended periods, particularly at night, as well as for combinations of agents with complementary mechanisms of action. Due to the ineffectiveness of current therapies to suppress cough episodes in many patients, the combination of an expectorant to facilitate productive cough and an extended-release opiate to decrease cough frequency may bring incremental and clinically desirable results.

The current strategies for cough suppression should be reassessed through the implementation of controlled clinical trials in large populations. Evidence-based medicine should guide the development of novel treatments that can more effectively reduce the social and healthcare burden associated with acute cough.

## Competing interests

PD is a consultant to Boehringer Ingelheim, Reckitt-Benckiser Inc., Merck, Novartis, Procter & Gamble, and Schering-Plough, is on the speakers' bureau of Boehringer-Ingelheim and Pfizer, and has received research grants from Adams and Boehringer-Ingelheim.

GC has served as a speaker/consultant/advisory board member for Adams, Almirall, Boehringer-Ingelheim, Forest, Genentech, GlaxoSmithKline, Schering-Plough, Lilly, Novartis, Pfizer, and Teva. MG and GR report no competing interests to disclose. SS was an investigator and/or speaker for Pharmaxis, Abbott, Amgen, AstraZeneca, Boehringer Ingelheim, Genentech, Johnson & Johnson, Novartis, Sanofi-Aventis, Schering, Merck and Sepracor. BW has received consultant fees from Reckitt Benckiser Inc., and Boehringer-Ingelheim.

## Authors' contributions

This paper is the result of an active discussion, involving participation and interaction among all authors. Accordingly, no author was in charge of any particular section, and it is not possible to detail each individual contribution in every subject. PD was responsible for the overall design/structure of the article, as well as the coordination of the project and the rendering of the written product. All authors read and approved the final version of the manuscript.
